# Chronic kidney disease and its association with cataracts–A cross-sectional and longitudinal study

**DOI:** 10.3389/fpubh.2022.1029962

**Published:** 2022-12-07

**Authors:** Chun-Yen Huang, Jia-In Lee, Chia-Wen Chang, Yao-Hua Liu, Shu-Pin Huang, Szu-Chia Chen, Jiun-Hung Geng

**Affiliations:** ^1^Department of Emergency Medicine, Kaohsiung Municipal Siaogang Hospital, Kaohsiung Medical University Hospital, Kaohsiung Medical University, Kaohsiung, Taiwan; ^2^Department of Psychiatry, Kaohsiung Medical University Hospital, Kaohsiung, Taiwan; ^3^Department of Urology, Kaohsiung Medical University Hospital, Kaohsiung Medical University, Kaohsiung, Taiwan; ^4^Graduate Institute of Clinical Medicine, College of Medicine, Kaohsiung Medical University, Kaohsiung, Taiwan; ^5^Department of Internal Medicine, Kaohsiung Municipal Siaogang Hospital, Kaohsiung Medical University, Kaohsiung, Taiwan; ^6^Division of Nephrology, Department of Internal Medicine, Kaohsiung Medical University Hospital, Kaohsiung Medical University, Kaohsiung, Taiwan; ^7^Faculty of Medicine, College of Medicine, Kaohsiung Medical University, Kaohsiung, Taiwan; ^8^Research Center for Environmental Medicine, Kaohsiung Medical University, Kaohsiung, Taiwan; ^9^Department of Urology, Kaohsiung Municipal Siaogang Hospital, Kaohsiung, Taiwan

**Keywords:** chronic kidney disease, cataract, risk factors, epidemiology, longitudinal cohort, cross-sectional cohort, glomerular filtration rate

## Abstract

**Introduction:**

We aim to explore the association between chronic kidney disease (CKD) and cataracts.

**Methods:**

A total of 121,380 participants with adequate information collected from 29 community-based recruitment centers since 2008 were analyzed. The association between CKD and self-reported diagnosed cataracts was examined in a cross-sectional cohort and was validated in a longitudinal cohort of 25,263 participants without cataracts at baseline.

**Results and discussion:**

Of all participants, cataracts occurred in 503/1,947 (26%) and 10,464/119,433 (9%) subjects in the CKD and non-CKD groups, respectively. Multivariate logistic regression showed that CKD was significantly associated with a higher prevalence of self-reported diagnosed cataracts. In the validation cohort, a higher incidence of cataracts was also noted in the CKD group (65/317, 21%) compared to the non-CKD group (1,964/24,252, 8%) during a mean 47-month follow-up. After adjusting for confounders, subjects with CKD had a 1.498-fold higher risk of incident cataracts than those without CKD (95% confidence interval = 1.114 to 2.013, *p* value = 0.007). We found that CKD was associated with a higher prevalence of cataracts as well as incident cataracts, which suggests CKD patients and their primary physicians should be aware of this disease and can provide a clue for further exploration of the possible mechanisms and treatments.

## Introduction

Cataracts, or opacification of the lens, are a significant cause of blindness especially in middle to low-income countries. It is estimated that over 15.2 million people are blind and over 78.8 million people are moderate to severe vision impaired secondary to cataracts worldwide (1). Despite advances in cataract surgery, cataracts continue to be a public health and financial burden due to the extended life expectancy and growing population. In order to identify the reversible risk factors of cataracts, it is important to halt the disease's development.

Chronic kidney disease (CKD), defined as decreased kidney function or presence of kidney damage for 3 or more months, is associated with several diseases, including hypertension (2), diabetes mellitus (DM) (3), and cerebral vascular disease (4). Moreover, CKD shares common pathogenetic mechanisms with various eye diseases, such as microvascular dysfunction, epithelial dysfunction, oxidative stress an inflammation (5). Recent studies have examined the association between CKD and cataracts (6, 7), but lack evidence of large-scale cross-sectional and longitudinal cohort studies. Here, we present major evidence for the association between CKD and cataracts.

## Materials and methods

### Data source and study population

**Figure 1:**
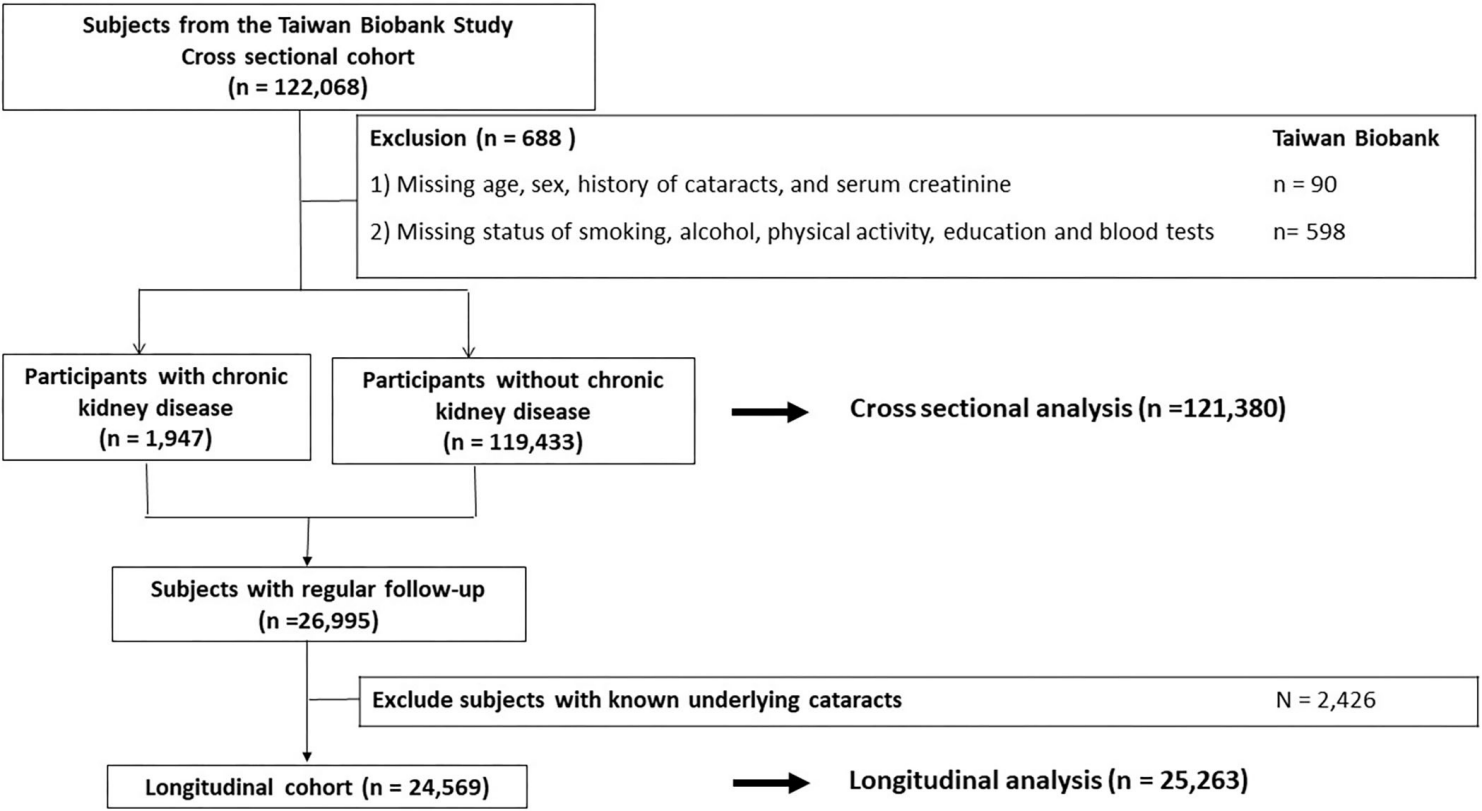
Study participants were classified by the presence of chronic kindey disease.

All participants were enrolled at 29 community-based recruitment centers in Taiwan since 2008. They were between 30 and 70 years old and had no history of malignancies when joining the cohort. Other detailed information has been described previously (8–10). In the cross-sectional cohort, a total of 121,380 participants with adequate information about age, sex, serum creatinine and history of cataracts, and possible confounders were analyzed (Figure 1). Then, a longitudinal cohort enrolled 25,263 participants without cataracts at baseline and with adequate long-term follow-up was used to validate the results of the cross-sectional analysis (Figure 1). The investigations followed the Declaration of Helsinki and participants signed a consent form. This study was approved by our institute (KMUHIRB-E(I)-20210058).

### Definition of CKD

In the present study, CKD is defined as an estimated glomerular filtration rate (eGFR) <60 ml/min/1.73 m^2^. Based on eGFR, we divided participants into CKD and non-CKD groups. Equation used to calculate eGFR (ml/min/1.73 m^2^) is [(140–age) × body weight / (0.814 × Serum creatinine in μmol/L)] × (0.85 if female).

### Self-reported diagnosed cataracts

Firstly, participants were asked, “Have you ever been diagnosed with cataracts?” Self-reported diagnosed cataracts were defined as participants answering “Yes” to this question. For those that have a past history of cataracts, they would be asked “Which eye was diagnosed with cataracts?” and “When were you diagnosed with cataracts?” Researchers would repeat these questions to ensure consistent responses.

### Variables and variables selection

All the variables in this study came from questionnaires, physical examination, and blood draws. The potential risk factors for the association between CKD and cataracts were identified through literature reviews, including age (11, 12), gender (13), body mass index (14), smoking status, alcohol status (15), education status, hypertension (including systolic and diastolic pressures) (16), diabetes mellitus (including fasting glucose), dyslipidemia (including total cholesterol and triglyceride), hemoglobin (17), albumin (18) and uric acid (19). To further identify the possible confounders, these variables were further investigated for their association with cataracts through univariate logistic regression, and then put into multivariate analysis if they were significant.

### Statistical analyses

In the present study, participants were classified as CKD and non-CKD groups. Their clinical characteristics were described as percentages for qualitative variables and mean ± standard deviation for quantitative variables. The differences between groups were calculated by using Pearson χ^2^ test or independent *t*-test, depending on the types of variables. To determine the association between the presence of CKD and cataracts, univariate and multivariate logistic regression analyses were used with odds ratios (ORs) and 95% confidence intervals (CIs). SPSS 20.0 (?IBM Corp, Armonk, NY, USA) and R version 3.6.2 (R Foundation for Statistical Computing, Wien, Austria) were two statistical tools we used and a *p*-value of <0.05 was regarded as statistically significant.

## Results

### Clinical characteristics of the study participants in the cross sectional cohort

The clinical characteristics for all participants, those with CKD and those without CKD are shown in Table 1. Of the 121,380 participants, 77,764 (64%) were female, and 1,947 (2%) and 119,433 (98%) were classified as CKD and non-CKD groups, respectively. The subjects with CKD were more likely to be male gender, older, had higher body mass index (BMI), systolic blood pressure (SBP) and diastolic blood pressure (DBP), and a higher prevalence of smoking, drinking, past history of hypertension, past history of DM, and past history of dyslipidemia compared to the men without CKD (Table 1). In addition, self-reported diagnosed cataracts occurred in 503/1,947 (26%) and 10,464/119,433 (9%) subjects in the CKD and non-CKD groups, respectively (Table 1).

**Table 1:** Clinical characteristics of the cross-sectional cohort (*n* = 121,380).

**Characteristics**	**Total** **(*N* = 121,380)**	**Chronic kidney disease** **(*N* = 1,947)**	**No chronic kidney disease** **(*N* = 119,433)**	***p*-value**
**Demographic data**				
Age, yr	50 ± 11	60 ± 8	50 ± 11	<0.001
Women, *n* (%)	77,764 (64)	1,183 (61)	77,000 (65)	<0.001
Body mass index, kg/m^2^	24.2 ± 3.8	25.8 ± 3.9	24.2 ± 3.8	<0.001
Smoke, ever, *n* (%)	33,073 (27)	771 (40)	32,302 (27)	<0.001
Alcohol status, ever, *n* (%)	10,331 (9)	276 (14)	10,055 (8)	<0.001
Education status, *n* (%)				<0.001
≦Elementary	6,407 (5)	278 (14)	6,129 (5)	
Middle to high school	44,617 (37)	808 (42)	43,809 (37)	
≧Collage	70,356 (58)	861 (44)	69,495 (58)	
Systolic blood pressure, mm Hg	120 ± 19	134 ± 21	120 ± 19	<0.001
Diastolic blood pressure, mm Hg	74 ± 11	79 ± 13	74 ± 11	<0.001
**Comorbidity**				
Hypertension, *n* (%)	14,849 (12)	944 (49)	13,905 (12)	<0.001
Diabetes mellitus, *n* (%)	6,258 (5)	464 (24)	5,794 (5)	<0.001
Dyslipidemia, *n* (%)	9,018 (7)	446 (23)	8,572 (7)	<0.001
**Laboratory data**				
Hemoglobin, g/dl	13.8 ± 1.6	13.6 ± 1.9	13.8 ± 1.6	0.002
Albumin, g/dl	4.5 ± 0.2	4.4 ± 0.3	4.5 ± 0.2	<0.001
Fasting glucose, mg/dl	96 ± 21	108 ± 36	96 ± 20	<0.001
Total cholesterol, mg/dl	196 ± 36	194 ± 41	196 ± 36	0.043
Triglyceride, mg/dl	116 ± 94	157 ± 134	115 ± 93	<0.001
Uric acid, mg/dL	5.4 ± 1.4	7.0 ± 1.8	5.4 ± 1.4	<0.001
eGFR, ml/min per 1.73 m^2^	103 ± 24	48 ± 14	104 ± 23	<0.001
Self-reported diagnosed cataracts	10,967 (9)	503 (25)	1,444 (9)	<0.001

eGFR, Estimated glomerular filtration rate.

### The association between CKD and prevalence of cataracts

To examine the association between CKD and self-reported diagnosed cataracts, logistic regression analyses were used. Univariate logistic regression showed that age, sex, BMI, smoking, drinking, educational status, SBP, DBP, past history of hypertension, past history of DM, past history of dyslipidemia, serum hemoglobin, albumin, fasting glucose, total cholesterol, triglyceride, and CKD were significantly associated with self-reported diagnosed cataracts (Table 2). After adjustment for confounders, age, sex, BMI, smoking, DBP, past history of hypertension, past history of DM, past history of dyslipidemia, serum albumin and CKD were still significantly related to cataracts (Table 2). Compared with those in the non-CKD group, the odds of cataracts in the CKD group were 3.627 (95% CI = 3.271–4.023, *p*-value = <0.001) and 1.335 (95% CI = 1.189–1.500, *p*-value = <0.001) in univariate and multivariate analyses, respectively. We further divided participants by age (<60 years old and ≧60 years old) and the results remained the same (Supplementary Table 1).

**Table 2:** Odds ratios for self-reported diagnosed cataracts in univariable and multivariable binary logistic analysis in the cross-sectional cohort (*n* = 121,380).

**Variables**	**Non-adjusted odds ratio (95% CI)**	***P***	**Adjusted odds ratio (95% CI)**	***P***
Age (per 1 year)	1.172 (1.168–1.175)	<0.001	1.169 (1.165–1.173)	<0.001
Male (vs. female)	0.857 (0.822–0.893)	<0.001	0.706 (0.660–0.756)	<0.001
Body mass index (per 1 kg/m^2^)	0.994 (0.988–0.999)	0.017	0.979 (0.972–0.986)	<0.001
Smoking status, ever (vs. never)	0.828 (0.791–0.867)	<0.001	1.106 (1.038–1.180)	0.002
Alcohol status, ever (vs. never)	0.918 (0.854–0.987)	0.021	0.973 (0.894–1.060)	0.534
Education status, middle to high school (vs. ≦Elementary)	0.374 (0.351–0.400)	<0.001	0.929 (0.865–0.997)	0.042
Education status, ≦Collage (vs. ≧Elementary)	0.251 (0.235–0.267)	<0.001	1.064 (0.990–1.144)	0.091
Systolic blood pressure (per 1 mm Hg)	1.020 (1.019–1.021)	<0.001	0.999 (0.997–1.000)	0.123
Diastolic blood pressure (per 1 mm Hg)	1.004 (1.002–1.006)	<0.001	0.996 (0.993–0.999)	0.005
Hypertension, yes (vs. no)	2.623 (2.502–2.751)	<0.001	1.139 (1.075–1.207)	<0.001
Diabetes mellitus, yes (vs. no)	3.775 (3.552–4.012)	<0.001	1.755 (1.618–1.904)	<0.001
Dyslipidemia, yes (vs. no)	2.862 (2.707–3.026)	<0.001	1.378 (1.293–1.470)	<0.001
Hemoglobin (per 1 g/dl)	0.986 (0.974–0.998)	0.025	0.981 (0.961–1.000)	0.050
Albumin (per 1 g/dl)	0.393 (0.362–0.427)	<0.001	0.848 (0.766–0.938)	0.001
Fasting Glucose, mg/dl	1.010 (1.009–1.010)	<0.001	1.001 (1.000–1.002)	0.087
Total cholesterol (per 1 mg/dl)	1.003 (1.002–1.003)	<0.001	1.000 (0.999–1.000)	0.202
Triglyceride (per 1 mg/dl)	1.000 (1.000–1.001)	<0.001	1.000 (1.000–1.000)	0.922
Uric acid (per 1 mg/dl)	1.013 (0.999–1.027)	0.067	-	-
Chronic kidney disease, yes (vs. no)	3.627 (3.271–4.023)	<0.001	1.335 (1.189–1.500)	<0.001

CI, Confidence interval. Adjusted by age, gender, body mass index, smoking status, alcohol status, education status, systolic blood pressure, diastolic blood pressure, hypertension, diabetes mellitus, dyslipidemia, hemoglobin, albumin, fasting glucose, total cholesterol, triglyceride, and chronic kidney disease.

### The association between CKD and the development of cataracts

To further validate the association between CKD and cataracts, the risk of CKD for developing cataracts was evaluated in a longitudinal subgroup of 24,569 participants who had no history of cataracts at baseline (Table 3). In this cohort, 15,876 (65%) were female and incident cataracts occurred in 2,029 (8%) participants during a mean 47-month follow-up. The subjects with CKD were more likely to be male gender, older, had higher BMI, SBP, and DBP, and a higher prevalence of smoking, drinking, past history of hypertension, past history of DM, and past history of dyslipidemia compared to the men without CKD (Table 3). A higher incidence of cataracts was noted in the CKD group (65/317, 21%) compared to the non-CKD group (1,964/24,252, 8%). Univariate logistic regression showed that age, sex, smoking, drinking, SBP, past history of hypertension, past history of DM, past history of dyslipidemia, serum albumin, fasting glucose, total cholesterol, triglyceride, and CKD were significantly associated with incident cataracts (Table 4). In multivariate analysis, the risk of developing cataracts was still higher in participants with older age, past history of DM, past history of dyslipidemia, and CKD (Table 4). Compared with those in the non-CKD group, the odds of cataracts in the CKD group were 2.927 (95% CI = 2.220–3.860, *p*-value = <0.001) and 1.498 (1.114–2.013, *p*-value = 0.007) in univariate and multivariate analyses, respectively. We further divided participants by age (<60 years old and >60 years old) and the results for those ≧60 years old were similar to the entire cohort; however, for those <60 years old, the relationship between CKD and cataracts was not statistically significant (Supplementary Table 2).

**Table 3:** Clinical characteristics of the longitudinal cohort (*n* = 24,569).

**Characteristics**	**Total** ** (*N* = 24,569)**	**Chronic kidney disease** ** (*N* = 317)**	**No chronic kidney disease** ** (*N* = 24,252)**	***p*-value**
Age, yr	50 ± 10	59 ± 8	50 ± 10	<0.001
Women, *n* (%)	15,876 (65)	131 (41)	15,745 (65)	<0.001
Body mass index, kg/m^2^	24.1 ± 3.6	25.9 ± 4.2	24.1 ± 3.6	<0.001
Smoke, ever, *n* (%)	6,009 (25)	117 (37)	5,892 (24)	<0.001
Alcohol status, ever, *n* (%)	2,065 (8)	41 (13)	2,024 (8)	0.004
Education status, *n* (%)				<0.001
≦Elementary	1,612 (7)	56 (18)	1,556 (6)	
Middle to high school	10,934 (44)	135 (42)	10,799 (45)	
≧Collage	12,023 (49)	126 (40)	11,897 (49)	
Systolic blood pressure, mm Hg	117 ± 18	130 ± 20	117 ± 18	<0.001
Diastolic blood pressure, mm Hg	73 ± 11	78 ± 12	73 ± 11	<0.001
Hypertension, *n* (%)	2,906 (12)	138 (44)	2,768 (11)	<0.001
Diabetes mellitus, *n* (%)	1,044 (4)	48 (15)	996 (4)	<0.001
Dyslipidemia, *n* (%)	1,636 (7)	60 (19)	1,576 (7)	<0.001
Hemoglobin, g/dl	13.8 ± 1.6	13.8 ± 1.8	13.7 ± 1.6	0.290
Albumin, g/dl	4.6 ± 0.2	4.5 ± 0.3	4.6 ± 0.2	<0.001
Fasting glucose, mg/dl	96 ± 19	104 ± 32	95 ± 19	<0.001
Total cholesterol, mg/dl	195 ± 35	199 ± 39	195 ± 35	0.090
Triglyceride, mg/dl	113 ± 83	148 ± 82	113 ± 83	<0.001
Uric acid, mg/dL	5.5 ± 1.4	7.3 ± 1.7	5.5 ± 1.4	<0.001
eGFR, ml/min per 1.73 m^2^	103 ± 24	50 ± 11	104 ± 23	<0.001
Follow-up, months	47 ± 14	48 ± 15	47 ± 14	0.338

eGFR, Estimated glomerular filtration rate.

**Table 4:** Odds ratios for self-reported diagnosed cataracts in univariable and multivariable binary logistic analysis in the longitudinal cohort (*n* = 24,569).

**Variables**	**Non-adjusted odds ratio (95% CI)**	***P***	**Adjusted odds ratio (95% CI)**	***P***
Age (per 1 year)	1.137 (1.129–1.144)	<0.001	1.143 (1.135–1.152)	<0.001
Male (vs. female)	0.793 (0.718–0.874)	<0.001	0.667 (0.584–0.763)	<0.001
Body mass index (per 1 kg/m^2^)	0.999 (0.987–1.012)	0.890	-	-
Smoking status, ever (vs. never)	0.739 (0.659–0.828)	<0.001	0.940 (0.808–1.093)	0.421
Alcohol status, ever (vs. never)	0.787 (0.658–0.941)	0.009	0.925 (0.757–1.131)	0.447
Education status, middle to high school (vs. ≦Elementary)	0.709 (0.403–1.246)	0.232	-	-
Education status, ≦Collage (vs. ≧Elementary)	0.766 (0.431–1.363)	0.365	-	-
Systolic blood pressure (per 1 mm Hg)	1.015 (1.013–1.018)	<0.001	0.993 (0.991–0.996)	<0.001
Diastolic blood pressure (per 1 mm Hg)	1.001 (0.997–1.005)	0.524	-	-
Hypertension, yes (vs. no)	2.012 (1.791–2.261)	<0.001	1.026 (0.895–1.175)	0.715
Diabetes mellitus, yes (vs. no)	2.698 (2.292–3.176)	<0.001	1.427 (1.165–1.748)	0.001
Dyslipidemia, yes (vs. no)	2.111 (1.828–2.437)	<0.001	1.254 (1.070–1.470)	0.005
Hemoglobin (per 1 g/dl)	0.978 (0.950–1.006)	0.121	-	-
Albumin (per 1 g/dl)	0.521 (0.428–0.634)	<0.001	1.149 (0.917–1.440)	0.226
Fasting glucose, mg/dl	1.007 (1.006–1.009)	<0.001	1.000 (1.000–1.001)	0.954
Total cholesterol (per 1 mg/dl)	1.003 (1.002–1.004)	<0.001	0.998 (0.997–1.000)	0.014
Triglyceride (per 1 mg/dl)	1.001 (1.000–1.001)	0.005	1.000 (1.000–1.001)	0.141
Uric acid (per 1 mg/dl)	0.989 (0.958–1.002)	0.512	-	-
Chronic kidney disease, yes (vs. no)	2.927 (2.220–3.860)	<0.001	1.498 (1.114–2.013)	0.007

CI, Confidence interval. Adjusted by age, gender, smoking status, alcohol status, systolic blood pressure, hypertension, diabetes mellitus, dyslipidemia, albumin, fasting glucose, total cholesterol, triglyceride, and chronic kidney disease.

## Discussion

In this study, we found that the risk of cataracts was higher in CKD patients (OR = 1.335; 95% CI, 1.189–1.500) in the cross-sectional cohort after adjusting for confounders. Furthermore, we examined 24,569 participants who had mean follow up periods of 47-months from 29 community-based recruitment centers without a history of cataracts at baseline and found that the incidence of developing cataracts (OR = 1.498; 95% CI, 1.114–2.013) is significantly higher in patients with CKD. To the best of our knowledge, this is the first large-scale cross-sectional and longitudinal study to investigate the relationship between CKD and risk of cataracts.

There were several previous cohort studies evaluating the association between CKD and cataracts but the results varied. Klein et al. (20) conducted a prospective study over a 5 years period in 1988 and found that renal function abnormalities were not significantly associated with incidence of cataracts after controlling for age and gender. Huynh et al. (21) found that there were no significant effects of CKD stage on the incidence of any type of cataract in agreement with that of Klein et al. However, they reported increased odds of incident cataract surgery in patients with moderate to severe renal dysfunction younger than 60 years of age. The authors attributed this effect to additional factors involving patients undergoing cataract surgery. Klein et al. (22) further investigated the relationship between cystatin C and the 15-year incidence of age-related cataracts in 2008. They concluded with contradictory results that increasing cystatin C levels was associated with an increased risk of age-related cataracts, especially cortical and posterior subcapsular subtypes. In recent years, Liu et al. (7) retrospectively analyzed 1-million subjects from a Taiwanese health insurance database. They found that the risk of cataract was higher in CKD group (adjusted hazard ratio 1.86) than non-CKD group and an even higher risk of cataracts in end stage renal disease group (adjusted hazard ratio 2.33), which indicated more severe renal dysfunction increased the risk of cataracts. However, the accuracy of the diagnosis of CKD and cataracts recorded by the International Classification of Disease Codes (ICD) has been questioned. The study performed by Rim et al. (23) also concluded that end stage renal disease patients who started hemodialysis were more likely to undergo cataract surgery. The results of our study were consistent with Liu et al. but constructed on more solid evidence by combining cross-sectional and longitudinal cohorts. In addition, patients diagnosed with CKD were based on eGFR in our study compared to Liu et al. which used ICDs.

Age-related cataracts comprised the vast majority of cataracts, but the risk factors were different from early-onset cataracts. Some known risk factors for early-onset cataracts included trauma, ultraviolet-B exposure, steroid use, diabetes mellitus and myotonic dystrophy, but not CKD (24–26). In our longitudinal cohort, we divided participants by age (<60 years old and ≧60 years old) and found that the relationship between CKD and cataracts was not statistically significant in those <60 years old, supporting the previous results.

In addition to CKD, we found that diabetes mellitus and dyslipidemia were associated with an increasing risk of cataracts. Our investigation was similar to the results of Liu et al. (7) in which diabetes and hyperlipidemia were independent risk factors associated with cataracts but contradictory results of alcohol use. Moreover, several previous studies have been performed which verified the association between diabetes and cataracts (27–31), and dyslipidemia and cataracts (32–34).

Proven by Liu et al. (7) the risk for cataracts increased with the severity of renal impairment. The results indicated that prevention of CKD progression and early eye screening programs might decrease the incidence of cataracts. As for diabetes, previous studies showed better glycaemic control was associated with a lower incidence of cataracts (34–36). A meta-analysis concluded that statin therapy could protect against cataract formation (37). Our study further validated the association between cataracts and CKD, diabetes mellitus and dyslipidemia which provided physicians perspectives on early screening and prevention of cataracts in these populations.

Several mechanisms were proposed to contribute to the relationship between CKD and cataracts. Urea sequestration in the lens with subsequent water accumulation and formation of osmotic cataracts was one of the possible mechanisms (38). Another possible mechanism was oxidative stress in CKD patients forming advanced glycation end products (AGEs) and leading to cataract formation (39, 40). AGEs acting on endothelial cells could lead to apoptosis, cell cycle arrest and generation of proinflammatory cytokines. These factors cause insolubility of lens proteins, accumulation of fluorescent products in the lens nucleus and the lens eventually losing its transparency.

This is the first study combining cross-sectional and longitudinal cohorts to analyze the association between CKD and risk of cataracts. Additionally, the diagnosis of CKD was based on eGFR, not by coding. Despite these strengths, the present study has several limitations. Firstly, data on cataracts were obtained from questionnaires, thus the diagnosis of cataracts lacked objective comprehensive ophthalmic examinations, and we did not have patient data for cataract subtypes and grades, nor for surgical modality and surgery-related complications; however, this substitution has been validated in other large-scale studies (41–43). Secondly, the database did not include other risk factors of cataracts, such as myopia, eye trauma, steroid use, ultraviolet light exposure and uveitis but recruited as many confounders as possible to strengthen the results. Thirdly, this study only involved Asians, so the results might not be representative of other ethnicities. Fourthly, we were unable to analyze the association between severity of CKD and cataracts because we had fewer than 100 subjects with end-stage renal disease. Fifthly, a rigorous definition of CKD should include follow-up eGFR data over 3-months and evidence of proteinuria, as opposed to a single eGFR at the time of admission. However, this substitution has been validated in other large-scale studies (44, 45). Finally, information regarding genetic testing was lacking, but most of the subjects in this study did not have congenital cataracts (46).

## Conclusion

We found that CKD was associated with a higher prevalence of cataracts as well as incident cataracts, which suggests CKD patients and their primary physicians should be aware of this disease and can provide a clue for further exploration of the possible mechanisms and treatments.

## Data availability statement

The data analyzed in this study is subject to the following licenses/restrictions: The data underlying this study are from the Taiwan Biobank. Due to restrictions placed on the data by the Personal Information Protection Act of Taiwan, the minimal data set cannot be made publicly available. Data may be available upon request to interested researchers. Please send data requests to S-CC (scarchenone@yahoo.com.tw), Division of Nephrology, Department of Internal Medicine, Kaohsiung Medical University Hospital, Kaohsiung Medical University.

## Ethics statement

The studies involving human participants were reviewed and approved by Institutional Review Board of Kaohsiung Medical University Hospital (KMUHIRB-E(I)-20210058). The patients/participants provided their written informed consent to participate in this study.

## Author contributions

Conceptualization, methodology, software, data curation, writing—review and editing, visualization, and project administration: J-HG. Formal analysis: J-IL and J-HG. Investigation: C-WC and Y-HL. Validation, resources, supervision, and funding acquisition: S-PH and S-CC. Writing–original draft preparation: C-YH. All authors have read and agreed to the published version of the manuscript.
