# Supplementary material for: Chronic kidney disease and its association with cataracts–A cross-sectional and longitudinal study
Source: Front Public Health. 2022 Dec 7;10:1029962. doi: 10.3389/fpubh.2022.1029962 (PMC9771793; doi:10.3389/fpubh.2022.1029962)
Supplement: Supplementary file 1 [file Data_Sheet_1.docx]

**Supplementary Table 1.** **Relative risk for the prevalence of self-reported diagnosed cataracts in the cross-sectional cohort divided by age (n=121,380)**

| Variables | No. of cataracts / No. of subjects (%) | Non-adjusted odds ratio (95% CI) | P | Adjusted odds ratio (95% CI) | P |
| --- | --- | --- | --- | --- | --- |
| **Total** |  |  |  |  |  |
| No Chronic kidney disease | 10,464/119,433 (9) | 1.000 (reference) |  | 1.000 (reference) |  |
| Chronic kidney disease | 503/1,947 (26) | 3.627 (3.271 to 4.023) | <0.001 | 1.335 (1.189 to 1.500) | <0.001 |
| **< 60 years old** |  |  |  |  |  |
| No Chronic kidney disease | 3,861/95,690 (4) | 1.000 (reference) |  | 1.000 (reference) |  |
| Chronic kidney disease | 122/847 (14) | 4.002 (3.295 to 4.861) | <0.001 | 1.825 (1.481 to 2.248) | <0.001 |
| **≧60 years old** |  |  |  |  |  |
| No Chronic kidney disease | 6,603/23,743(28) | 1.000 (reference) |  | 1.000 (reference) |  |
| Chronic kidney disease | 381/1,100 (35) | 1.376 (1.211 to 1.562) | <0.001 | 1.222 (1.067 to 1.401) | 0.004 |

CI = Confidence interval.

Adjusted by age, gender, body mass index, smoking status, alcohol status, education status, systolic blood pressure, diastolic blood pressure, hypertension, diabetes mellitus, dyslipidemia, hemoglobin, albumin, fasting glucose, total cholesterol, triglyceride, and chronic kidney disease.

**Supplementary Table 2. Relative risk for the incidence of self-reported diagnosed cataracts in the longitudinal cohort divided by age (n=24,569)**

| Variables | No. of cataracts / No. of subjects (%) | Non-adjusted odds ratio (95% CI) | P | Adjusted odds ratio (95% CI) | P |
| --- | --- | --- | --- | --- | --- |
| **Total** |  |  |  |  |  |
| No Chronic kidney disease | 1,964/24,252 (8) | 1.000 (reference) |  | 1.000 (reference) |  |
| Chronic kidney disease | 65/317 (21) | 2.927 (2.220 to 3.860) | <0.001 | 1.498 (1.114 to 2.013) | 0.007 |
| **< 60 years old** |  |  |  |  |  |
| No Chronic kidney disease | 1,056/20,097 (5) | 1.000 (reference) |  | 1.000 (reference) |  |
| Chronic kidney disease | 22/171 (13) | 2.662 (1.694 to 4.183) | <0.001 | 1.596 (0.996 to 2.556) | 0.052 |
| **≧60 years old** |  |  |  |  |  |
| No Chronic kidney disease | 908/4,155 (22) | 1.000 (reference) |  | 1.000 (reference) |  |
| Chronic kidney disease | 43/146 (30) | 1.493 (1.038 to 2.147) | 0.031 | 1.493 (1.025 to 2.174) | 0.037 |

CI = Confidence interval.

Adjusted by age, gender, smoking status, alcohol status, systolic blood pressure, hypertension, diabetes mellitus, dyslipidemia, albumin, fasting glucose, total cholesterol, triglyceride, and chronic kidney disease.
